# MiR-532-3p suppresses colorectal cancer progression by disrupting the ETS1/TGM2 axis-mediated Wnt/β-catenin signaling

**DOI:** 10.1038/s41419-019-1962-x

**Published:** 2019-09-30

**Authors:** Chuncai Gu, Jianqun Cai, Zhijun Xu, Shiming Zhou, Liangying Ye, Qun Yan, Yue Zhang, Yuxin Fang, Yongfeng Liu, Chenge Tu, Xinke Wang, Juan He, Qingyuan Li, Lu Han, Xin Lin, Aimin Li, Side Liu

**Affiliations:** 1 Guangdong Provincial Key Laboratory of Gastroenterology, Department of Gastroenterology, Nanfang Hospital, Southern Medical University, Guangzhou, 510515 China; 2 Department of General Surgery, Nanfang Hospital, Southern Medical University, Guangdong Provincial Engineering Technology Research Center of Minimally Invasive Surgery, Guangzhou, 510515 China

**Keywords:** Colorectal cancer, Oncogenes

## Abstract

The expression panel of plasma microRNA defined miR-532-3p as a valuable biomarker for colorectal adenoma (CRA). However, its expression pattern and function in colorectal cancer (CRC) have remained unclear. The present study investigated the expression levels of miR-532-3p and found that it was in situ downregulated both in CRA and CRC. Moreover, it functioned as a sensitizer for chemotherapy in CRC by inducing cell cycle arrest and early apoptosis via its activating effects on p53 and apoptotic signaling pathways. In addition, miR-532-3p was found to restrain cell growth, metastasis, and epithelial–mesenchymal transition (EMT) phenotype of CRC. A study on the mechanism behind these effects revealed that miR-532-3p directly binds to 3′UTR regions of ETS1 and TGM2, ultimately repressing the canonical Wnt/β-catenin signaling. Further investigation showed that TGM2 was transcriptionally regulated by ETS1 and ETS1/TGM2 axis served as a vital functional target of miR-532-3p in suppressing CRC progression. To conclude, miR-532-3p mimics could act as potential candidate for molecular therapy in CRC through inactivation of the canonical Wnt/β-catenin signaling and enhancement of chemosensitivity.

## Introduction

Colorectal cancer (CRC) represents a major health burden worldwide. It is a lethal disease and both genetic and environmental factors play a role in its etiology^1^. Despite the rapid progress in systemic cancer therapies, CRC is associated with a high mortality rate, which is largely attributed to late clinical diagnosis and high levels of recurrence and epigenetic alterations, along with transcriptional changes in non-coding RNAs^2^.

MicroRNAs (miRNAs) are a class of small non-coding RNAs that regulate gene expression by binding to the 3′ untranslated region (3′UTR)^3^. Several miRNAs, such as miR-494, miR-21, miR-196b-5p, and miR-203, have been verified to be involved in the stepwise progression of adenomas, oncogenesis, and promotion of cell migration or drug resistance in CRC. The earlier studies highlight the importance of the utility of miRNAs as novel predictive markers or potential therapeutic targets for CRC^4–8^. According to the plasma profile of microRNA obtained during a precise screening of colorectal adenoma, microRNA-532 (miR-532) was identified as one of the eight miRNAs for distinguishing polyps with high accuracy^9^. With context to malignancies, miR-532 has been reported as a prognostic biomarker for bladder cancer. Moreover, it has been shown to be associated with the Warburg effect in ovarian cancer^10,11^. Although miR-532 has been reported to be dysregulated in adenoma, its expression pattern and molecular function in CRC remain undefined.

In the present study, we report miR-532-3p, a mature sequence of miR-532, to be originally downregulated in CRC. The exogenous transfection of miR-532-3p remarkably promoted apoptosis and inhibited proliferation and invasion properties of CRC both in vivo and in vitro. The E26 oncogene homolog 1 (ETS1), a member of the ETS family of transcription factors (TFs) known to promote cellular growth and migration^12,13^, and transglutaminase 2 (TGM2), a well-known apoptosis attenuator, are both direct targets of miR-532-3p in CRC. In addition, TGM2 has been reported to be transcriptionally activated by ETS1 and inhibits apoptosis and associated with chemotherapy stress in CRC via the activation of Wnt/β-catenin signaling. Our results demonstrated the loss of miR-532-3p in CRC and the consequent suppression of the Wnt/β-catenin signaling via directly inhibiting the ETS1/TGM2 axis, providing a possible explanation for the observed chemotherapy resistance and potential therapeutic targets for CRC.

## Results

### MiR-532-3p is synchronously downregulated in colorectal adenoma and CRC

**Fig. 1:**
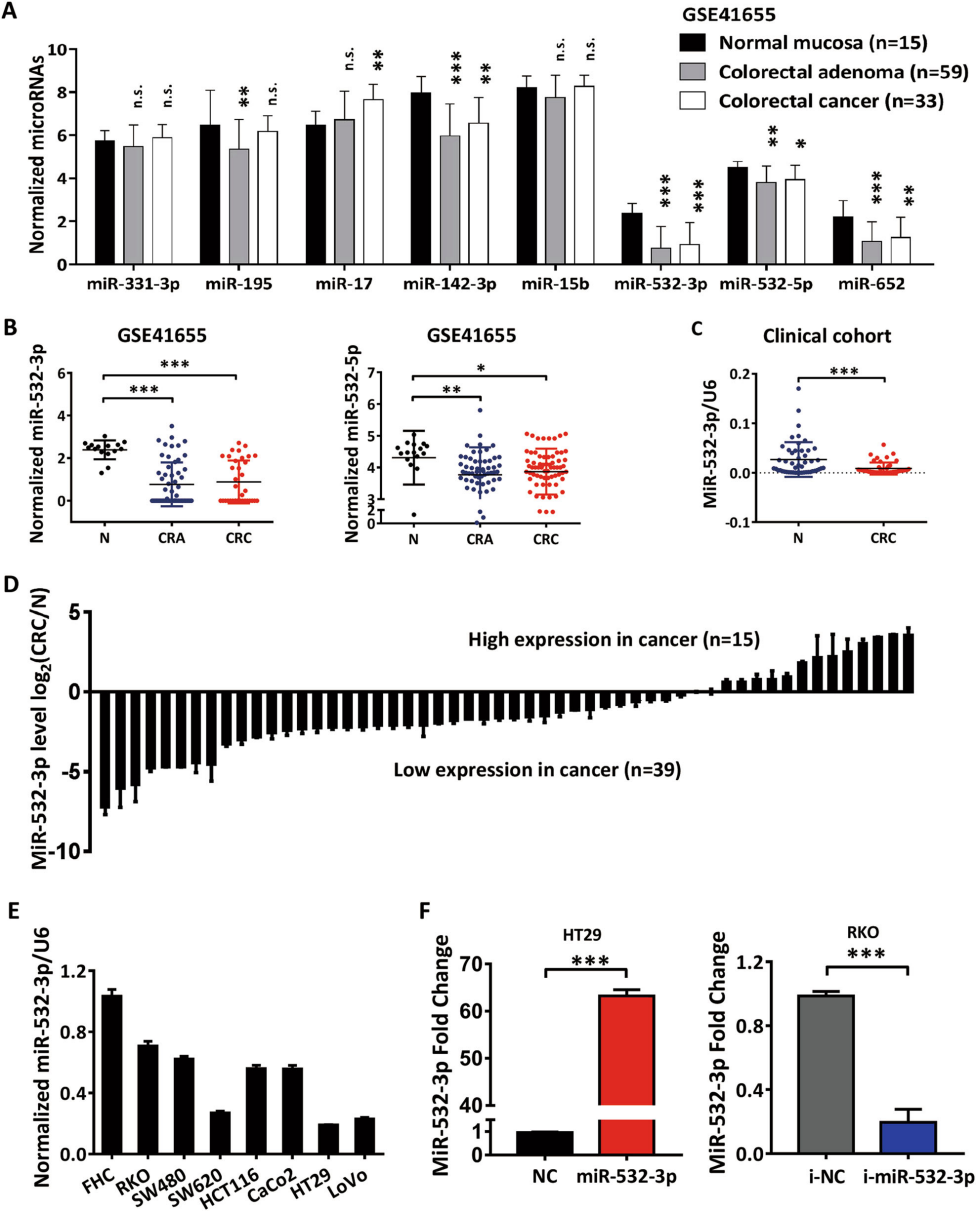
The downregulation of MiR-532-3p in colorectal cancer. **a** The expression patterns of eight miRNAs in normal colorectal mucosa (N), colorectal adenoma (CRA), and colorectal adenocarcinoma (CRC) tissues with a public expression profiling dataset GSE41655. **b** The normalized miRNAs expression levels of miR-532-3p and miR-532-5p in GSE41655 dataset. **c** Expression of miR-532-3p was verified in 54 pairs of CRC tissues from a clinical cohort, which was normalized against an endogenous U6 RNA control. **d** Relative miR-532-3p expression in CRC compared with that in the paired normal colorectal mucosa, the fold change was normalized with log_2_(CRC/N). **e** Expression of miR-532-3p in seven colorectal cancer cell lines and a colorectal mucosal cell line FHC. **f** Detection of miR-532-3p in HT29 cells transfected with miR-532-3p mimics and RKO cells with miR-532-3p inhibitors by qPCR. *p < 0.05, **p < 0.01, ***p < 0.001, n.s. non-significant

A screening project for colorectal adenoma (CRA) defined miR-532-3p as one of the eight miRNA panel biomarkers, along with miR-331, miR-195, miR-17, miR-142-3p, miR-15b, miR-532, and miR-652, to distinguish polyps from controls^9^. This was verified using the public CRA and CRC expression dataset (GSE41655). We found that both miR-532-3p and miR-532-5p, along with miR-142-3p and miR-652, were downregulated during the progression of CRA and eventually aggressive colorectal cancer (Fig. 1a). Due to the greater diversity observed in the expression of miR-532-3p (Fig. 1b), we confirmed this alteration using real-time quantitative PCR (qPCR) analysis in paired cancer tissues and normal colorectal mucosal tissues, which demonstrated a significant depletion of miR-532-3p in CRC tissues (Fig. 1c, d). In order to detect the function of miR-532-3p in CRC, we studied its expression in seven human colorectal cancer cell lines (HT29, SW480, Caco2, HCT116, LoVo, SW620, and RKO) and a normal colon epithelial cell line (FHC) and found the expression of miR-532-3p to be relatively lower in CRC cell lines than that in FHC cells (Fig. 1e). We selected HT29, having the lowest expression of miR-532-3p, and RKO, which had the highest expression, to be transfected with miR-532-3p mimics or inhibitors separately, with successful confirmation by qPCR (Fig. 1f). Cell lines stably overexpressing miR-532-3p (HT29-LV-miR-532-3p) or knock-downed (RKO-LV-i-miR-532-3p) were established by lentivirus infection.

### MiR-532-3p enhances p53-induced cell cycle arrest and apoptosis of CRC

**Fig. 2:**
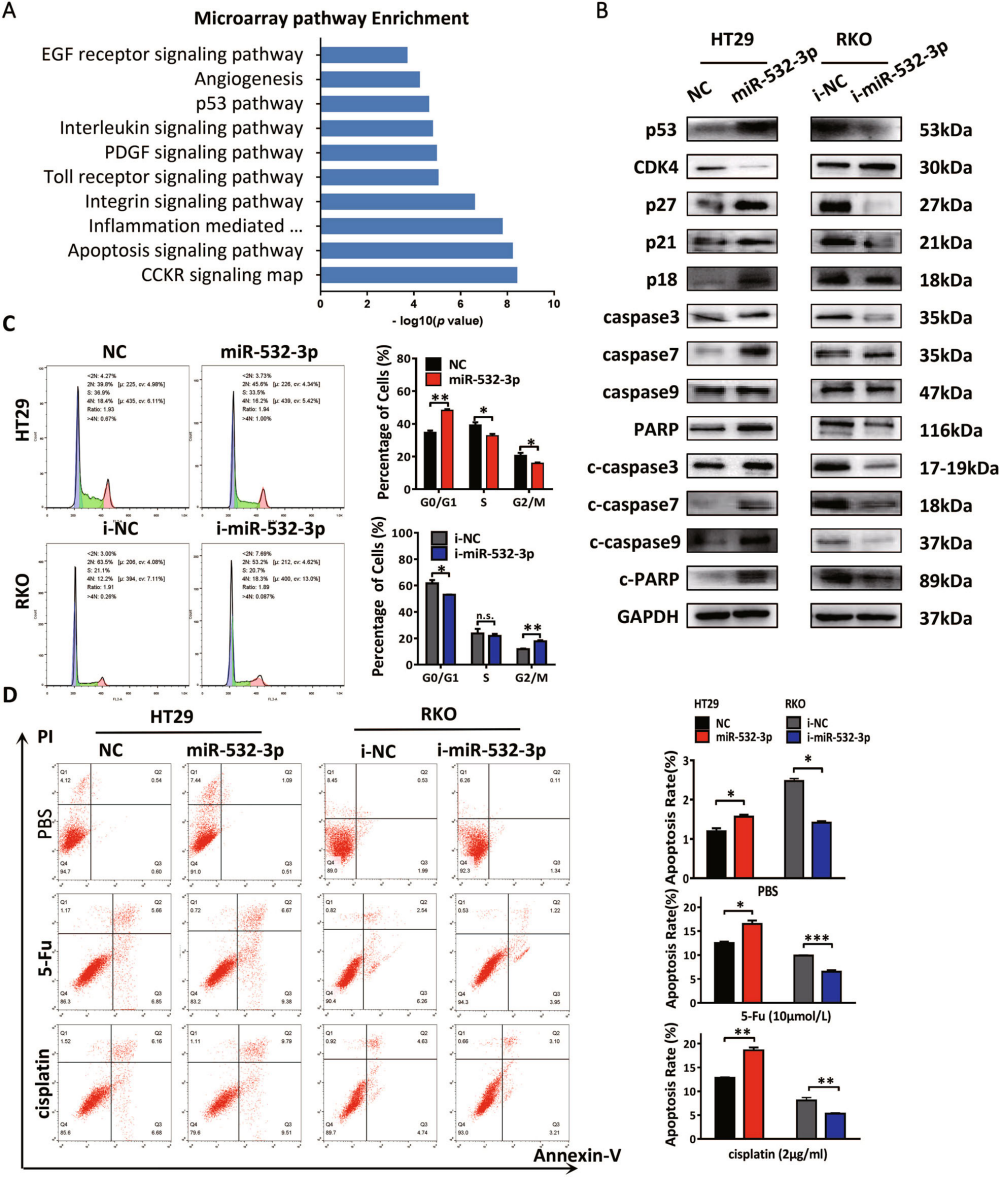
MiR-532-3p enhances p53-induced cell cycle arrest and apoptosis of CRC. **a** Panther Pathway enrichment of significant altered genes in HT29-miR-532-3p cells and top ten pathways have been shown. **b** MiR-532-3p-induced activation of cell cycle arrest and apoptosis signaling in CRC cells. **c** Cell cycle analysis showed that miR-532-3p-induced cell cycle arrest in HT29 cells and miR-532-3p inhibitors could accelerate the transition in RKO cells. **d** Transfection of miR-532-3p mimics could induce apoptosis in HT29 after treatment with 5-Fluorouracil (5-Fu) or cisplatin. Further, miR-532-3p inhibitors could enhance apoptosis resistance in RKO cells. *p < 0.05, **p < 0.01, ***p < 0.001, n.s. non-significant

In order to determine the potential functions of miR-532-3p in CRC, we analyzed the gene expression in HT29-LV-miR-532-3p using the Affymetrix microarray and performed pathway enrichment of significantly altered genes. Specifically, the analysis results revealed significant alteration of p53 and apoptosis signaling pathways induced by overexpression of miR-532-3p, as well as related pathways such as inflammation and integrin signaling (Fig. 2a). Further, p53 plays an important role in promoting tumor apoptosis and inhibiting cell growth via regulating the cellular apoptotic pathways and cell cycle checkpoints^14^. We detected the expression of p53-related cell cycle proteins, such as cyclin kinase inhibitors including p53, p21, and p18, and cyclin-dependent kinase 4 (CDK4) and apoptosis-related proteins using western blot. Activation of p53/p21 and p18 was induced by miR-532-3p mimics in HT29 cells, which could inhibit cell cyclin kinases such as CDK4 and promote cycle arrest. Inversely, miR-532-3p inhibitors decreased the expression of p53/p21 and p18, resulting in elevated CDK4 and accelerated cell cycle transition. Moreover, the results showed that the transfection of HT29 cells with miR-532-3p mimics could induce elevation of initiator caspases such as caspase 9, functional caspases such as caspase 3 and caspase 7, and DNA damage repair enzyme PARP, as well as the activation of apoptosis proteins, including cleaved-caspase (c-caspase) 9, c-caspase 3, c-caspase 7, and c-PARP. On the contrary, miR-532-3p inhibitors resulted in the decrease of cleaved-caspase proteins in RKO cells, indicating the specific function of miR-532-3p in promoting caspase-dependent apoptosis in CRC (Fig. 2b, Supplementary Fig. 1A). We thus performed cell cycle analysis to determine the effect of miR-532-3p on cell cycle transformation, which revealed miR-532-3p to induce cell cycle arrest in HT29 cells, whereas miR-532-3p inhibitors resulted in the accelerated cell cycle transition in RKO cells (Fig. 2c). To further determine whether miR-532-3p could induce apoptosis of CRC, cell apoptosis was studied using flow cytometry. The results revealed that miR-532-3p overexpression elevated the apoptosis rate of HT29 cells (Fig. 2d). Moreover, it showed that the exogenous transfection with miR-532-3p mimics significantly sensitized HT29 cells to chemotherapy by elevating early apoptosis of HT29 cells treated with a specific concentration of 5-fluorouracil or cisplatin. Moreover, treatment with miR-532-3p inhibitors promoted tolerance to chemotherapy in RKO cells (Fig. 2d). Based on these results, we determined that miR-532-3p might enhance the chemosensitivity in CRC by activating cell cycle arrest and apoptosis.

### MiR-532-3p suppresses cell proliferation and migration of CRC

**Fig. 3:**
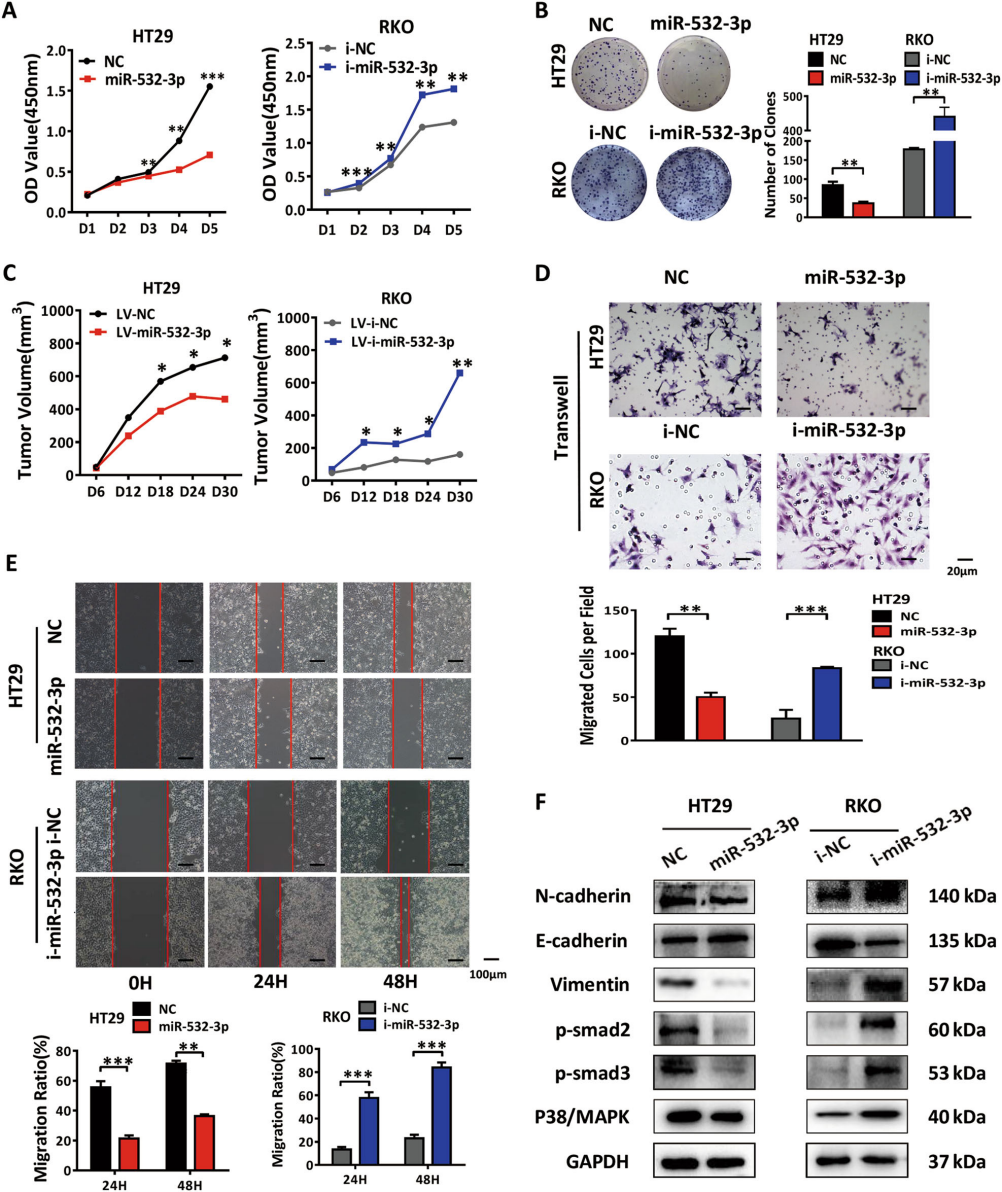
MiR-532-3p suppresses cell proliferation and migration of CRC. **a** MiR-532-3p mimics decreased the cell growth and the inhibitors restored the proliferation in HT29 and RKO cells by CCK-8. **b** Clone formation assay showed a decrease or increase in the proliferation of HT29 and RKO cells after transfection with miR-532-3p mimics and inhibitors as indicated. **c** Subcutaneous tumor growth curve of HT29 cells and RKO cells infected with NC, miR-532-3p, i-NC, and i-miR-532-3p lentivirus as indicated. **d** The transwell assay for HT29-NC, HT29-miR-532-3p, RKO-i-NC, RKO-i-miR-532-3p groups. **e** The wound-healing assay in HT29 and RKO cells transfected with miR-532-3p mimics or inhibitors as indicated. **f** Western blot analysis of EMT markers and Smad, MAPK proteins in HT29 and RKO cells. *p < 0.05, **p < 0.01, ***p < 0.001

**Fig. 4:**
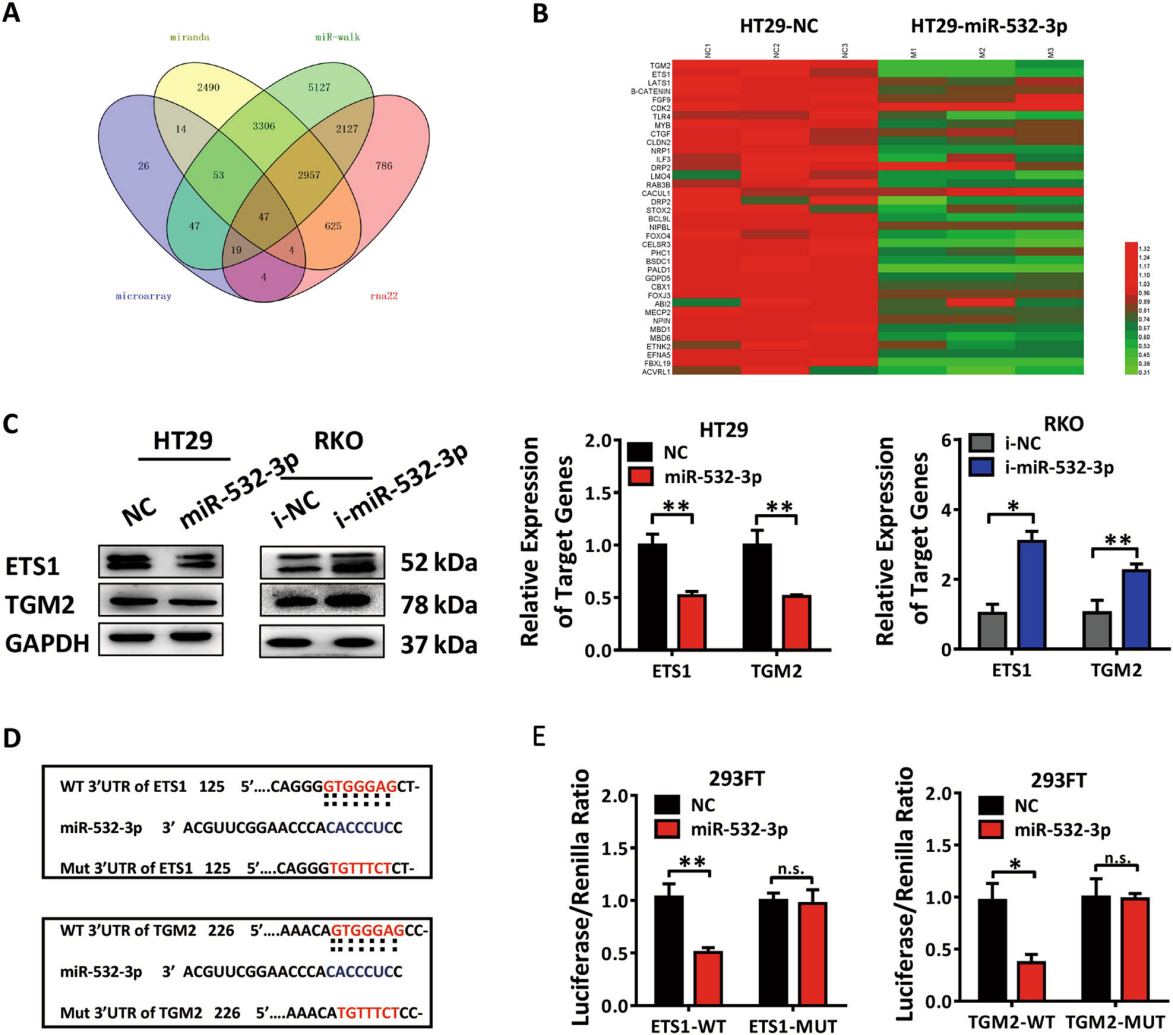
ETS1 and TGM2 as the direct targets of miR-532-3p. **a** The comparison among different miRNA target prediction engines (MiRanda, miR-walk, and rna22) combined with microarray data. **b** QPCR verification of the potential target genes of miR-532-3p in HT29 cells transfected with NC, miR-532-3p mimics. ETS1 and TGM2 were both significantly downregulated in HT29-miR-532-3p group. **c**. Western blot analysis and qPCR analysis of ETS1 and TGM2 in HT29 or RKO cells transfected with miR-532-3p mimics or inhibitors. **d** Bioinformatics analysis revealed the 3′UTR region of ETS1 and TGM2 contained binding sites of the seed sequence of miR-532-3p. **e** Dual-luciferase reporter assay illustrating the binding of miR-532-3p and ETS1 or TGM2 in 293FT cells. *p < 0.05, **p < 0.01, n.s. non-significant

A close relationship between proliferation and cell cycle regulation has been described in a number of studies^15^. To further demonstrate whether miR-532-3p-induced p53 pathway affected the growth of CRC, we assessed the proliferation ability of cancer cells in vitro. According to the results of CCK-8 assay, HT29 cells displayed sluggish growth from day 3 after transfection with miR-532-3p mimics, and RKO cells exhibited accelerated growth after treatment with miR-532-3p inhibitors (Fig. 3a). Similar results were obtained in the clone formation assay, with an obvious decrease and increase in the number of clones found in the HT29-miR-532-3p and RKO-i-miR-532-3p groups, respectively (Fig. 3b). The proliferation of CRC cells was subsequently measured in vivo by inoculating HT29-LV-miR-532-3p and RKO-LV-i-miR-532-3p cells subcutaneously into nude mice. We observed a time-dependent decelerated growth of tumors in HT29-LV-miR-532-3p group (Fig. 3c, Supplementary Fig. 1B). Immunohistochemistry (IHC) detection of Ki-67 in xenograft tumors revealed lower expression of Ki-67 in the HT29-LV-miR-532-3p group compared with the control group (Supplementary Fig. 1C). On the contrary, the RKO-LV-i-miR-532-3p group exhibited accelerated proliferation and greater tumor size, accompanied by the higher expression of Ki-67 (Fig. 3c, Supplementary Fig. 1B, C). These results determined that miR-532-3p inhibited CRC tumor growth both in vivo and in vitro, indicating that miR-532-3p could contribute to inhibition of progression of CRC growth. In addition, we detected miR-532-3p to inhibit the metastatic ability of CRC, as evident from significant suppression of migration of HT29 cells by transwell and wound-healing assays after treatment with miR-532-3p mimics, contrary to miR-532-3p inhibitors in RKO cells (Fig. 3d, e). Western blot analysis revealed suppressed expression of mesenchymal markers (N-cadherin and vimentin) and elevated expression of epithelial markers (E-cadherin) after treatment of HT29 cells with miR-532-3p mimics accompanied with inactivation of Smad and p38/MAPK signaling (Fig. 4f, Supplementary Fig. 1D). Contrary results were obtained in RKO cells after treatment with miR-532-3p inhibitors.

### ETS1 and TGM2 are both functional targets of miR-532-3p

The primary function of miRNAs in modifying cellular life is posttranscriptional suppression of target genes. Based on the data obtained from multiple miRNA target prediction engines (MiRanda, miR-walk, and rna22) and microarray data of HT29-LV-miR-532-3p cells on interactions between miRNAs and the genes with decreased expression (fold change < 0.66), we obtained 47 potential target genes of miR-532-3p (Fig. 4a). After filtration and qPCR verification, we determined ETS1 and TGM2 as two efficient genes to be closely correlated with the aggressive phenotype of CRC (Fig. 4b). ETS1, the founder member of the ETS-domain family, is a vital transcription factor (TF) that regulates a wide variety of biological processes, including cell growth, migration, and differentiation^16^. TGM2, a membrane enzyme involved in protein cross-linking and cell adhesion to fibronectin, has been reported to be related to cancer stem cell survival and tumor formation in multiple types of cancers^17^. The detection with qPCR and western blot revealed a negative correlation between the expression of miR-532-3p and ETS1, TGM2 in CRC cell lines (Supplementary Fig. 2A). Moreover, the depletion of ETS1 and TGM2 proteins was observed in HT29-LV-miR-532-3p cells, contrary to the results obtained in RKO-LV-i-miR-532-3p cells (Fig. 4c, Supplementary Fig. 2B). Inhibitory effects of miR-532-3p on ETS1 and TGM2 expression were verified in vivo with IHC staining in tumors formed in HT29-LV-miR-532-3p and RKO-LV-i-miR-532-3p cells (Supplementary Fig. 2C).

In order to determine the regulatory function of miR-532-3p in targeting ETS1 and TGM2, we performed computer-based sequence analysis (MiRanda) to acquire potential targeting positions and found binding regions of miR-532-3p located in the 3′UTR of ETS1 and TGM2 (Fig. 4d). The fragments of ETS1 and TGM2 3′UTR regions (ETS1-WT and TGM2-WT) and their corresponding mutant counterparts (ETS1-MUT and TGM2-MUT) were subcloned separately into a firefly luciferase reporter plasmid (GV272), followed by transfection into 293FT cells together with *Renilla* reporter plasmid and miR-532-3p mimics or NC for dual-luciferase reporter assay. Notably, firefly luciferase activity of both ETS1 and TGM2 3′UTRs was suppressed by co-transfection with miR-532-3p mimics, verifying ETS1 and TGM2 as the direct targets of miR-532-3p (Fig. 4e).

### ETS1 activates Wnt/β-catenin pathway by directly regulating TGM2 transcription and inducing CRC progression

**Fig. 5:**
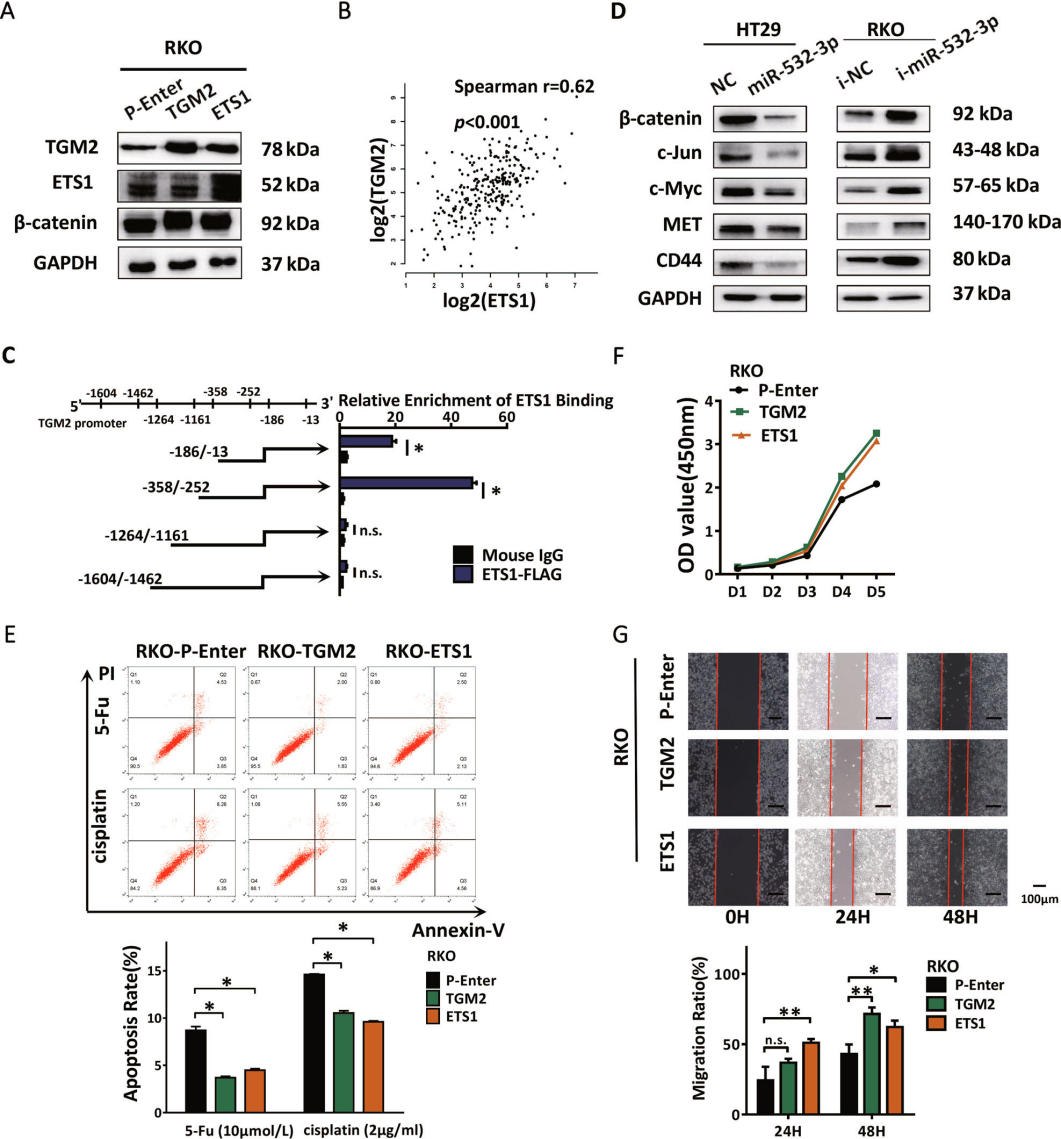
ETS1 activates Wnt/β-catenin pathway by directly regulating TGM2 transcription and induces CRC progression. **a** Western blot analysis of ETS1, TGM2, β-catenin in RKO cells transfected with TGM2, ETS1 plasmids, and P-Enter used as control. **b** Spearman’s correlation analysis showing a positive correlation between ETS1 and TGM2 mRNA levels according to TCGA datasets. **c** Chip-qPCR analysis of ETS1-binding regions in TGM2 promoter showing the important sites including −186/−13 and −358/−252. **d** Western blot analysis of β-catenin and downstream molecules of Wnt/β-catenin signaling in HT29-miR-532-3p cells and RKO-i-miR-532-3p cells. **e** Apoptosis analysis of RKO-P-Enter, RKO-ETS1, RKO-TGM2 cells after treatment with 5-Fu, cisplatin. **f** The CCK-8 assay was performed to detect the effect of ETS1 and TGM2 in the proliferation ability of RKO cells. **g** Wound-healing assay evaluating the motility of RKO-P-Enter, RKO-ETS1, RKO-TGM2 cells. *p < 0.05, **p < 0.01, n.s. non-significant

We next assessed the function of ETS1 and TGM2 in the progression of CRC. For this, we transfected the constructed plasmids into RKO cells separately and checked the efficiency using western blot (Fig. 5a, Supplementary Fig. 2D). Interestingly, the expression of TGM2 was upregulated after ETS1 transfection. We also performed an expression analysis of ETS1 and TGM2 with TCGA colorectal cancer datasets and verified that the expression level of ETS1 positively correlated with that of TGM2 (Fig. 5b). To understand the reason behind the promotion of TGM2 expression by ETS1 and the transcript regulatory effect of ETS1 in cancer, full-length TGM2 promoter was analyzed and potential TF binding sequences of ETS1 with abundant binding sites were predicted as shown by bioinformatics analysis website (LASAGNA-Search). The ChIP-qPCR analysis was subsequently performed to determine the binding sequence of ETS1 in RKO cells transfected with Flag-tagged-ETS1 plasmids. The fragmented TGM2 promoters were detected with qPCR to verify the potential ETS1-binding sites and among these, two recognition sites (−358 ~ −252, −186 ~ −13), specifically enriched by anti-Flag antibody compared with the IgG control, indicated TGM2 to be a direct target of transcript factor ETS1 in CRC (Fig. 5c).

TGM2 has previously been reported to promote nuclear accumulation of β-catenin by interacting with FZD7 and FN, whereas ETS1 has been implicated in the Wnt/β-catenin signaling in various cancers^18–21^. Western blot analysis of RKO cells revealed an elevation of β-catenin accompanied by an upregulation of ETS1 and TGM2 (Fig. 5a). Therefore, Spearman’s correlation analysis with TCGA datasets was performed, which showed the expression of β-catenin to be positively correlated with both ETS1 and TGM2, indicating regulatory functions of ETS1 and TGM2 in β-catenin overexpression in CRC (Supplementary Fig. 2E). The results showed elevated expression of β-catenin with the accumulation of ETS1 and TGM2. We speculated that miR-532-3p might regulate the expression of β-catenin via its modulation of ETS1 and TGM2. To verify this, we detected the protein levels in HT29-LV-miR-532-3p cells, and the result revealed suppressed function of miR-532-3p on β-catenin and the canonical targets of Wnt/β-catenin signaling, including c-Jun, c-Myc, CD44, and MET, which could be promoted by miR-532-3p inhibitors in RKO cells (Fig. 5d, Supplementary Fig. 2F). Previous microarray results have shown enrichment of the p53 pathway in HT29-LV-miR-532-3p cells. The Wnt/β-catenin signaling plays a critical role in defining progressive properties of cancer cells, including with various driver signaling in CRC such as p53 pathway^22^. We hypothesize miR-532-3p to act as a vital upstream inhibitor of the Wnt/β-catenin signaling via the ETS1/TGM2 axis and propose its involvement in inducing p53 activation and restraining aggressive phenotypes of CRC. In relation to the contribution of the ETS1/TGM2 axis in the invasive properties of CRC and 5-Fu or cisplatin in inducing apoptosis, the ETS1/TGM2 axis was found to decrease early apoptosis and promote chemotherapy resistance (Fig. 5e). Both CCK-8 and wound-healing assays reported comparable cell growth and motility induced by both ETS1 and TGM2 (Fig. 5f, g).

### MiR-532-3p restrains Wnt/β-catenin signaling via suppression of ETS1/TGM2 axis

**Fig. 6:**
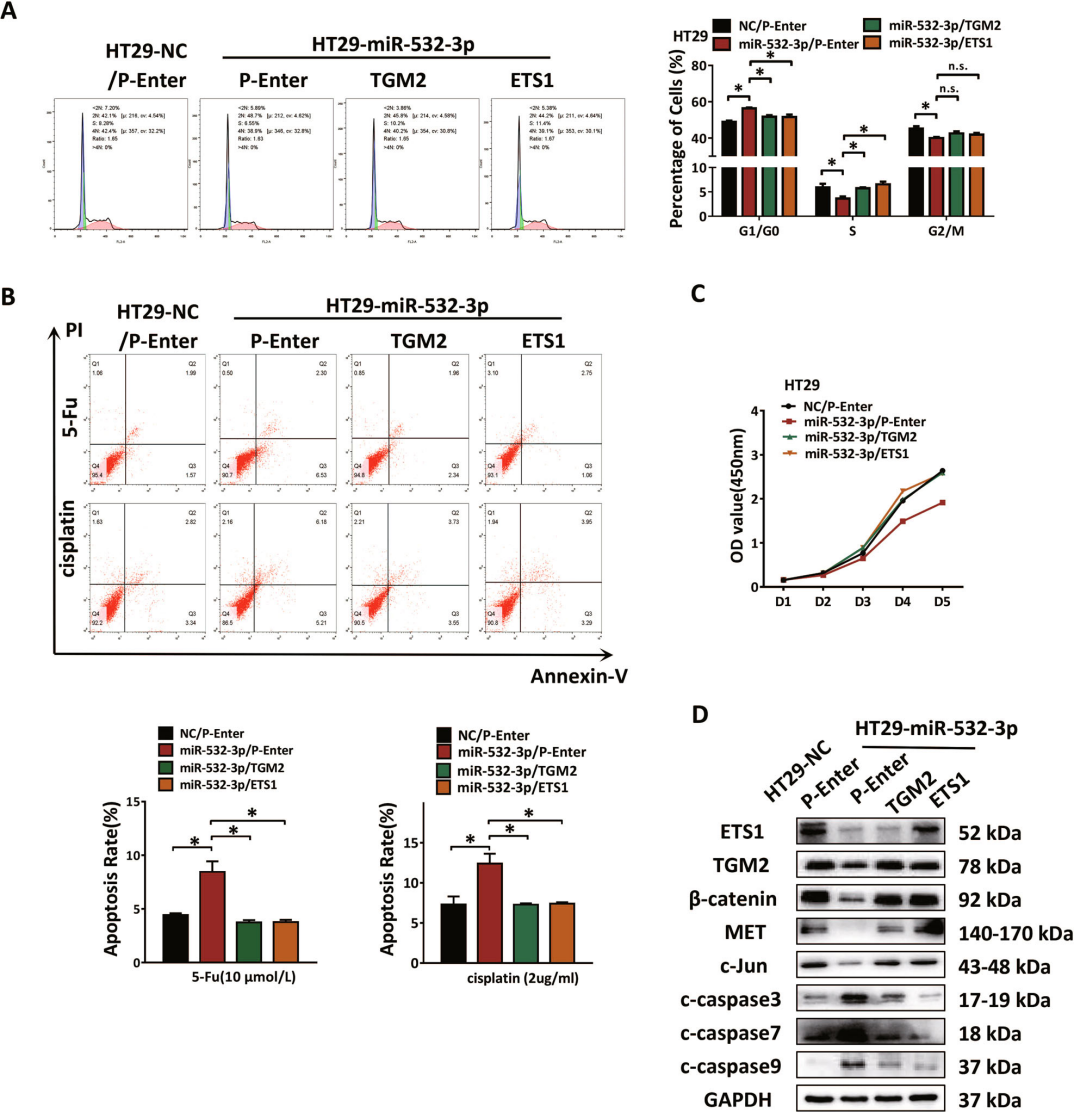
MiR-532-3p restrains Wnt/β-catenin signaling via suppression of ETS1/TGM2 axis. **a** MiR-532-3p-induced G0/G1 cell cycle arrest and ETS1 and TGM2 rescued the transition in HT29 cells. **b** ETS1 or TGM2 could rescue HT29-miR-532-3p cells from early apoptosis induced by 5-Fu and cisplatin. **c** Proliferation ability was determined with CCK-8 assay in HT29 cells; and ETS1 and TGM2 could elevate the cell growth by the suppression of miR-532-3p. **d** Western blot analysis of ETS1, TGM2, β-catenin, downstream molecules of β-catenin, and caspase proteins in HT29 cells after co-transfection with miR-532-3p mimics, ETS1, and TGM2 plasmids. *p < 0.05, n.s. non-significant

In order to confirm the role of the ETS1/TGM2 axis in miR-532-3p-mediated suppression of CRC progression, the cell cycle was analyzed. The results showed that rescuing the ETS1/TGM2 axis reversed the miR-532-3p-induced cell cycle arrest (Fig. 6a). MiR-532-3p associated chemotherapy-induced apoptosis in HT29 cells after treatment with 5-Fu or cisplatin was detected and rescuing the ETS1/TGM2 axis reversed this effect (Fig. 6b). The CCK-8 assay was then performed in HT29 cells that resulted in a recovery of cellular proliferation (Fig. 6c). Motility of RKO-miR-532-3p cells was enhanced by the ETS1/TGM2 axis as verified using the transwell and wound-healing assays (Supplementary Fig. 3A, B). Based on these results, we implied that the ETS1/TGM2 axis is essential in miR-532-3p inhibiting CRC progression. We further confirmed by protein detection in HT29-LV-miR-532-3p cells that the transfection of ETS1 or TGM2 plasmid could rescue the miR-532-3p-induced activation of Wnt/β-catenin pathway. The results indicated an essential involvement of the ETS1/TGM2 axis during the restraining process of miR-532-3p in Wnt/β-catenin signaling, including β-catenin, MET, and c-Jun with a decrease in the apoptosis-related proteins, such as c-caspase 3, c-caspase 7, and c-caspase 9, accompanied with rescued expression of ETS1 or TGM2 (Fig. 6d, Supplementary Fig. 3C).

## Discussion

The molecular mechanisms underlying the effects of miR-532-3p in progression of CRC have not been well described in the literature. Another mature sequence, miR-532-5p was reported to play dual roles in different types of cancers. Using the internal validation of our cohort and external verification of public GEO datasets, we determined the downregulation of miR-532-3p both in CRA and CRC. However, screening of plasma miRNA levels in CRA revealed miR-532-3p to be overexpressed in adenomas. This has been used in distinguishing certain cancer patients with high sensitivity^22^. Circulating miRNAs do not always correlate with tissue miRNAs. A notable example is miR-200c, its levels elevate in the plasma of patients with CRC along with a stage-dependent gradual decrease in primary CRC tissues^23^. In addition, a review on the potential serum miRNAs predictive models established in different clinical cohorts reported diverse plasma microRNA panels with great heterogeneity in different types of colorectal neoplasia^24,25^.

In the current study, we performed microarray detection and pathway enrichment in HT29 cells and validated the involvement of miR-532-3p in apoptotic pathways and activation of p53 signaling in CRC. MiR-532-3p was shown to retard G1/S cell cycle transition and cell growth of CRC both in vivo and in vitro, which was consistent with the proliferation inhibitory function of miR-532-5p in renal cancer^26^. However, in gastric cancer, exogenous miR-532-5p was found to accelerate the cell growth via targeting RUNX3 and acting as an oncogenic miRNA^27^. We attributed the inhibitory effect of miR-532-3p to its activation of canonical p53/p21 and p18/CDK4 pathways during the growth of CRC. Earlier studies have suggested an essential role of p53 in orchestrating cancer cell cycle arrest, senescence, and apoptosis as a function of its tumor-suppressor activity. Moreover, a panoply of miRNAs has been implicated in the p53 network^28^. We also observed that miR-532-3p promoted early apoptosis and enhanced the treatment effects of cisplatin and 5-Fu in CRC. This function was attributed to its regulation of the apoptotic pathway and elevation of downstream caspase proteins. In addition, miR-532-3p was shown to reverse invasion properties and EMT phenotype of CRC, similar to that of miR-532-5p in the modulation of TGFBR1^29^.

To check the versatility of miR-532-3p targets, we determined its major downstream targets and defined important roles of ETS1 and TGM2 in the progression of CRC. We accidentally found a close interaction between ETS1 and TGM2 through a transcription regulation. ETS1 was transcriptionally regulated via an ETS/AP-1 composite binding site, which could be enhanced by its autoregulation activity via stimulation by a growth factor or hypoxia during the progression of cancer. In the present study, we verified that miR-532-3p could suppress the expression of ETS1 via directly binding to the 3′UTR regions. In addition, we found an indirect regulation induced by miR-532-3p via its promotional effects on p53 activation, which has been reported as a direct repressor of ETS1 via a binding region around the nucleotide −30^30^. However, a further understanding of the major mechanism underlying the dual effects of miR-532-3p in ETS1 expression is required. With further verification of co-expression and transcript regulation of ETS1 in TGM2, we determined that TGM2 was regulated by miR-532-3p through the direct binding effects in the 3′UTR regions and indirect inhibitory effects on the transcriptional activity.

We reported that the overexpression of both ETS1 and TGM2 could induce aggressive phenotypes and drug resistance in CRC, at least in part, owing to their promoting effects on Wnt/β-catenin signaling. TGM2 has been reported to activate β-catenin overexpression via c-Src-dependent phosphorylation and its nuclear accumulation^31^. Here, we determined the overexpression of β-catenin to be induced by the ETS1/TGM2 axis in CRC, which could be restrained by miR-532-3p. Furthermore, we determined a promoting effect of miR-532-3p in CRC chemosensitivity via its inhibition of β-catenin and activation of p53. Multiple chemotherapies were verified to induce cell cycle arrest and early apoptosis by both inhibition of Wnt responsiveness and stabilization and activation of p53^32,33^. Moreover, the activation of Wnt/β-catenin and mutation or depletion of p53 were regarded as a fundamental process of adaptive resistance in CRC^34,35^. We made attempts to apply miR-532-3p as a small-molecule drug combined with chemotherapy in the treatment of CRC in vitro and found its decreased function in CRC proliferation and migration.

**Fig. 7:**
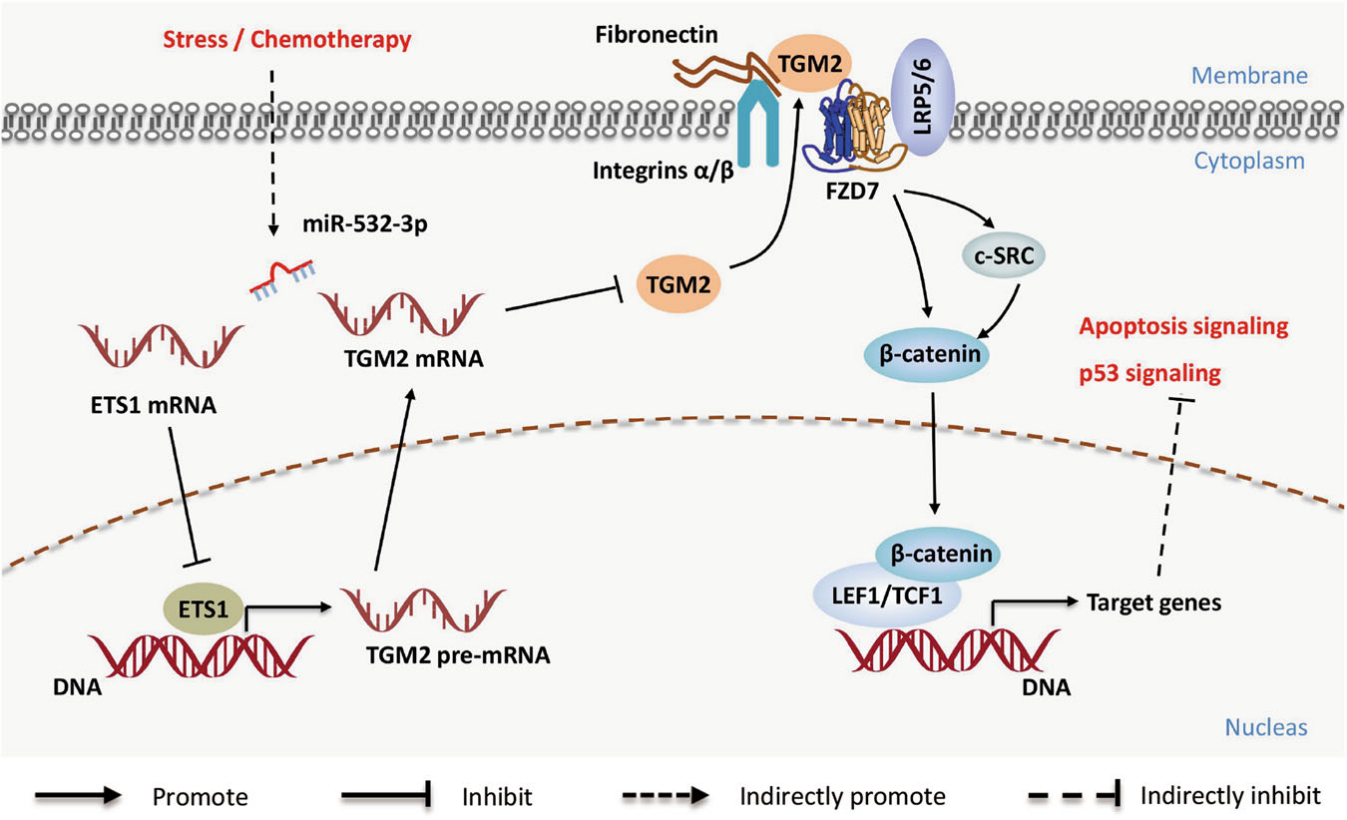
A hypothetical model illustrating that miR-532-3p directly targeted ETS1/TGM2 axis and activated p53 signaling and apoptosis signaling via the suppression of β-catenin and Wnt/β-catenin pathway downstream molecules. Meanwhile, it supposed a transcript regulation function of ETS1 in TGM2, indicating the indirect inhibition effect of miR-532-3p on TGM2

In summary, our study elucidated a complex molecular mechanism underlying the inhibitory effects of miR-532-3p in CRC progression. The ETS1/TGM2 axis-mediated Wnt/β-catenin signaling causes miR-532-3p to promote apoptosis and p53 pathway in CRC (Fig. 7). Extensive inhibitory effects on the ETS1/TGM2 axis made miR-532-3p an important inhibitor of cell growth and metastasis, and a chemotherapy sensitizer of CRC. These findings indicate that miR-532-3p functions as a tumor suppressor in CRC.

## Materials and methods

### Patients and specimens

The study involved a clinical cohort, with 54 pairs of fresh colorectal cancer tissues and normal colorectal mucosa tissues collected from patients with CRC who had undergone routine surgery at the Nanfang Hospital, Southern Medical University (Guangzhou, China) and volunteered to provide samples for research. The study was approved by the Ethics Committee of the Nanfang Hospital, Southern Medical University and data were analyzed anonymously.

### Cell lines and cell culture

Human colorectal cancer cell lines RKO, SW480, SW620, HCT116, CaCo2, HT29, LoVo, colorectal mucosal cell line FHC, and kidney cell 293FT were obtained from American Type Culture Collection (ATCC; Manassas, VA, USA). The cell lines were cultured in Dulbecco’s modified Eagle medium (DMEM) (Gibco, USA) with 10% fetal bovine serum (FBS; Gibco, USA) at 37 °C and 5% CO_2_.

### Reagents, lentivirus transduction, oligonucleotides, and plasmid transfections

Cisplatin (Qilu Pharmaceutical Ltd., Jinan, China) and 5-Fu (Xudong Pharmaceutical Co. Ltd., Shanghai, China) were separately diluted in phosphate-buffered saline (PBS) and stored at −20 °C. The cells were treated with 1, 3, 4, 5, 10, and 20 µg/mL cisplatin and 2.5, 5.0, 7.5, 10.0, 12.5, 15.0, and 20.0 µg/mL 5-Fu and incubated at 37 °C in 5% CO_2_ for 24 h. Half-maximal inhibitory concentration (IC_50_) was determined using the CCK-8 assay.

The full length of the hsa-miR-532-3p (miR-532-3p mimics) and inhibitors were chemically synthesized by GenePharma (China) and inserted into a lentivirus vector system (Obio Technology, China). The cells were infected with lentivirus particles and screened by puromycin. Transfection effects were verified with fluorescence intensity and qPCR detection. ETS1 and TGM2 plasmids were constructed by Vigenebio (Shandong, China), and P-Enter plasmid was used as a control. The sequences of plasmids or oligonucleotides are listed in Supplementary Table 1. Transfection was performed using Lipofectamine 3000 reagent following the manufacturer’s instructions (Invitrogen, USA).

### Microarray analysis

Total RNA was isolated from three biological replicated samples and cDNA was prepared according to the standard Affymetrix protocol. Affymetrix microarray detection and data standardization were performed by LongSee (Guangzhou, China).

### RNA isolation, quantitative real-time PCR, and western blot analysis

Total RNA was extracted from tissue samples and cultured cells using the Trizol reagent (Takara), and qPCR was performed using the PrimeScript RT reagent kit (Takara) and SYBR Premix Ex Taq (Takara, USA) according to the manufacturer’s instructions. GAPDH was used as an internal control to detect the mRNA expression. For the detection of miRNA, Mir-X^TM^ miRNA First-Strand Synthesis Kit (Takara, USA) was used for synthesizing cDNA from different types of miRNAs and U6 small nuclear RNA was used as an internal control. Data analysis was performed using the 2^–ΔΔCt^ method. Primers are listed in Supplementary Table 1.

Standard western blot procedure was performed and the protein bands were transferred onto a nitrocellulose membrane (Bio-Rad, USA). Non-fat milk (5%) was used for blocking and the membranes were incubated with primary antibodies against GAPDH (Proteintech, 1:1000), β-catenin (CST, 1:1000); TGM2 (CST, 1:1000); ETS1 (Abcam, 1:1000) overnight. After incubation with an appropriate secondary antibody, the protein bands were visualized using Immobilon ECL (Millipore).

### Cell count kit assay

Cells transfected with appropriate plasmids were seeded into a 96-well plate at 1000 cells per well, and cultured at 37 °C at 5% CO_2_ for 6 h. For cell count kit (CCK-8) assay, the cells were incubated with 10 μL of CCK-8 (Dojindo, Japan) for 2 h at 37 °C and their density was measured at a wavelength of 450 nm using the Paradigm Detection Platform (Beckman, CA, USA). The cells were further incubated for 5 days followed by the CCK-8 assay.

### Migration assays

The transwell assay was performed to test the migration of cells. Cells suspended with serum-free DMEM at a density of 5 × 10^4^ cells in 200 μL were placed into transwell chambers (Costar, USA) filled with 600 μL DMEM including 20% FBS in the lower chambers. After incubation at 37 °C for 24 h, the cells were fixed with 4% paraformaldehyde and stained with crystal violet solution (Sangon Biotech Co, China) for 2 min. The cells on the upper surface of the membranes were then removed, and cells on the lower surface were counted in five high-power fields under a light microscope. For the wound-healing assay, the cells were seeded into a 6-well plate and grown to 80% confluency. Three parallel “wounds” were made by scraping the cell monolayers with a pipette tip. The cells were then cultured with DMEM containing 2% FBS, and the migration of cells was observed every 12 h under an inverted microscope (Olympus, Japan).

### Apoptosis and cell cycle assays

Annexin V-FITC/PI Apoptosis Detection Kit (BD Biosciences, USA) was used to conduct apoptosis assay according to the manufacturer’s instructions. For cell cycle analysis, the cells were fixed with chilled 70% ethanol for 14 h at 4 °C and stained with PI/RNase staining buffer (BD Bioscience, USA) for detection.

### In vivo tumor growth assay

Four- to six-week-old athymic male BALB/c nu/nu mice were obtained from the Central Laboratory of Animal Science at Southern Medical University, and maintained at the Laboratory Animal Centre of Nanfang Hospital in a specific pathogen-free environment. A total of 5 × 10^6^ cells were infected with miR-532-3p-overexpressing lentivirus, or miR-532-3p-knockdown lentivirus was subcutaneously injected into the right flank of the nude mice (*n* *=* 6 per group). Tumors were measured with a caliper every 6 days after injection, and the mice were sacrificed 5 weeks later. Tumors were dissected and tumor volumes were calculated according to the formula: (length × width^2^)/2. Tumor samples were then fixed with paraffin for immunohistochemistry.

### Immunohistochemistry

Consecutive tumor sections (4 μm in thickness) were prepared and subjected to immunohistochemistry (IHC). After deparaffinization and antigen retrieval, sections were incubated with antibodies against TGM2 (CST, 1:200), ETS1 (Abcam, 1:200), and Ki-67 (Abcam, 1:400) overnight followed by incubation with an appropriate secondary antibody for 1 h. The sections were observed with the DAB chromogenic agent (ZSGB-BIO) under the light microscope.

### Luciferase reporter assay

Wild-type 3′UTR of TGM2 (TGM2-WT) and ETS1 (ETS1-WT) and their mutant segments were cloned and ligated into the GV272 vector. Further, 293FT cells were seeded into 24-well plates and grown to 80% confluency. These were then transfected with the appropriate plasmids together with a *Renilla* vector (Genechem, China). After 48 h, the cells were harvested and lysed to perform luciferase assay following the manufacturer’s instructions of the Dual-Luciferase Reporter Assay System (Promega, USA).

### Chromatin immunoprecipitation

Chromatin immunoprecipitation (ChIP) assay was performed according to the protocol of EZ-ChIP™ Chromatin Immunoprecipitation Kit (Millipore, USA) in RKO cells transfected with ETS1-FLAG-pEnter plasmids. Immunoprecipitation was performed using mouse monoclonal anti-FLAG antibody (Sigma, USA). To amplify the potential ETS1-binding sites in the promoter of TGM2, four pairs of specific primers were used for qPCR analysis (Supplementary Table 1).

### Bioinformatic analysis

The expression profiling data of miRNAs analyzed in the present study were downloaded from the TCGA database (http://cancergenome.nih.gov/) and the GEO database. KOBAS 3.0 server was used for pathway enrichment analysis according to the PANTHER pathway public datasets^36^. The correlation between ETS1 and TGM2, ETS1 and β-catenin, and TGM2 and β-catenin was analyzed using the colorectal adenocarcinoma datasets from TCGA via gene expression profiling interactive analysis (GEPIA)^37^. MiRNA target genes were predicted using MiRanda, miR-walk, rna22 databases.

### Statistical analysis

GraphPad Prism software (Version 5.0, Inc., San Diego, CA, USA) and SPSS software (Version 19.0; Abbott Laboratories, Chicago, IL) were used for statistical analysis. Data are expressed as mean ± standard error of mean unless otherwise stated. Student’s *t*-test was used to detect significance between the groups, and *χ*^2^ test was used for measuring the data. Analysis of variance (ANOVA) was used for determining differences among three or more groups followed by post-hoc analysis. Spearman’s correlation analysis was performed to detect the expression correlation.

## Supplementary information

Supplementary figure legends

Supplementary figure 1

Supplementary figure 2

Supplementary figure 3

Supplementary Table 1
