# Supplementary material for: MiR-532-3p suppresses colorectal cancer progression by disrupting the ETS1/TGM2 axis-mediated Wnt/β-catenin signaling
Source: Cell Death Dis. 2019 Sep 30;10(10):739. doi: 10.1038/s41419-019-1962-x (PMC6768886; doi:10.1038/s41419-019-1962-x)
Supplement: Supplementary file 1 — Supplementary figure legends [file 41419_2019_1962_MOESM1_ESM.docx]

Supplementary Figure 1

**A.** Gray analysis of protein detection with Western blot on miR-532-3p suppressing p53 and apoptosis pathway. **B.** The final volume of subcutaneous tumor in HT29-LV-NC, HT29-LV-miR-532-3p, RKO-LV-i-NC, RKO-LV-i-miR-532-3p groups, and volume of isolated tumor measured with ImageJ. **C.** Ki-67 staining of subcutaneous tumor in HT29-LV-NC, HT29-LV-miR-532-3p, RKO-LV-i-NC, RKO-LV-i-miR-532-3p group. **D.** Quantification of Western blot analysis of EMT markers and smad, MAPK proteins in HT29 and RKO cells. *p<0.05, **p<0.01, ***p<0.001.

**Supplementary Figure 2**

**A.** Western blot analysis of ETS1 and TGM2 and qPCR detection of miR-532-3p in HT29, LoVo, SW480, RKO cell lines. **B.** Quantification of western blot of ETS1 and TGM2 in HT29 or RKO cells transfected with miR-532-3p mimics or inhibitors. **C.** IHC staining of ETS1 and TGM2 in subcutaneous tumor of the HT29-LV-NC, HT29-LV-miR-532-3p, RKO-LV-i-NC, RKO-LV-i-miR-532-3p groups. **D.** Quantification of western blot of ETS1, TGM2, and β-catenin after transfection of ETS1 and TGM2 plasmids comparing with P-Enter. **E.** Spearman correlation analysis of ETS1 and β-catenin, TGM2 and β-catenin according to mRNA dataset from TCGA. **F.** Quantification of western blot of Wnt/β-catenin pathway downstream genes after transfection with miR-532-3p mimics in HT29 cells and inhibitors in RKO cells. *p<0.05, **p<0.01, ***p<0.001, n.s. non-significant.

.**Supplementary Figure 3**

**A.** Transwell assay was performed in RKO cells co-transfected with miR-532-3p mimics and ETS1 or TGM2 as indicated. **B.** Wound healing assay was performed in RKO cells co-transfected with miR-532-3p mimics and ETS1 or TGM2 as indicated. **C.** Quantification of western blot analysis of ETS1, TGM2, β-catenin, downstream molecules of β-catenin and caspase proteins in HT29 cells after co-transfection with miR-532-3p mimics and ETS1, TGM2 plasmids. *p<0.05, **p<0.01, ***p<0.001, n.s. non-significant.
