# Supplementary figures and images for: MiR-532-3p suppresses colorectal cancer progression by disrupting the ETS1/TGM2 axis-mediated Wnt/β-catenin signaling
Source: Cell Death Dis. 2019 Sep 30;10(10):739. doi: 10.1038/s41419-019-1962-x (PMC6768886; doi:10.1038/s41419-019-1962-x)

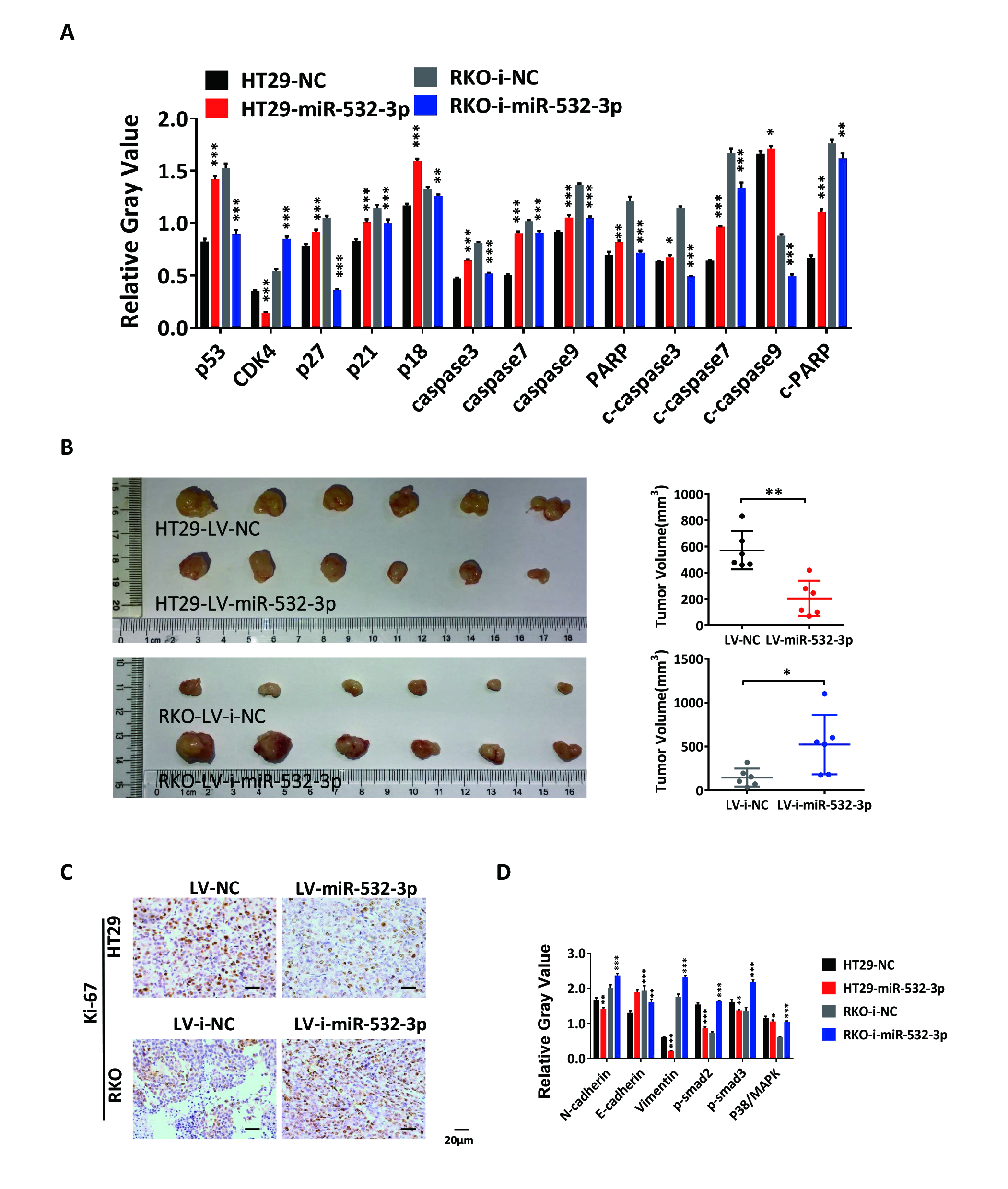

Supplement: Supplementary file 2 — Supplementary figure 1 [file 41419_2019_1962_MOESM2_ESM.tif]

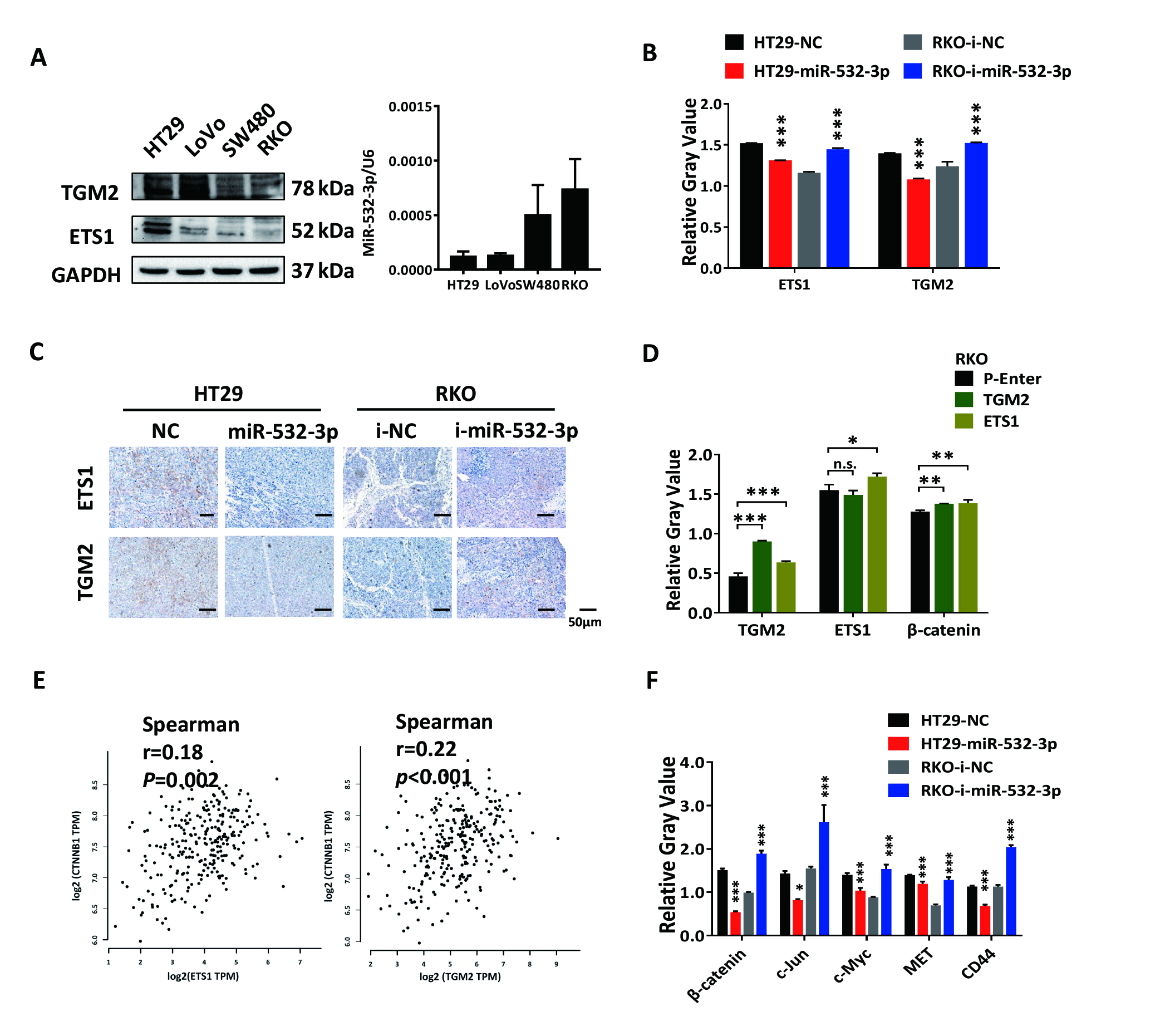

Supplement: Supplementary file 3 — Supplementary figure 2 [file 41419_2019_1962_MOESM3_ESM.tif]

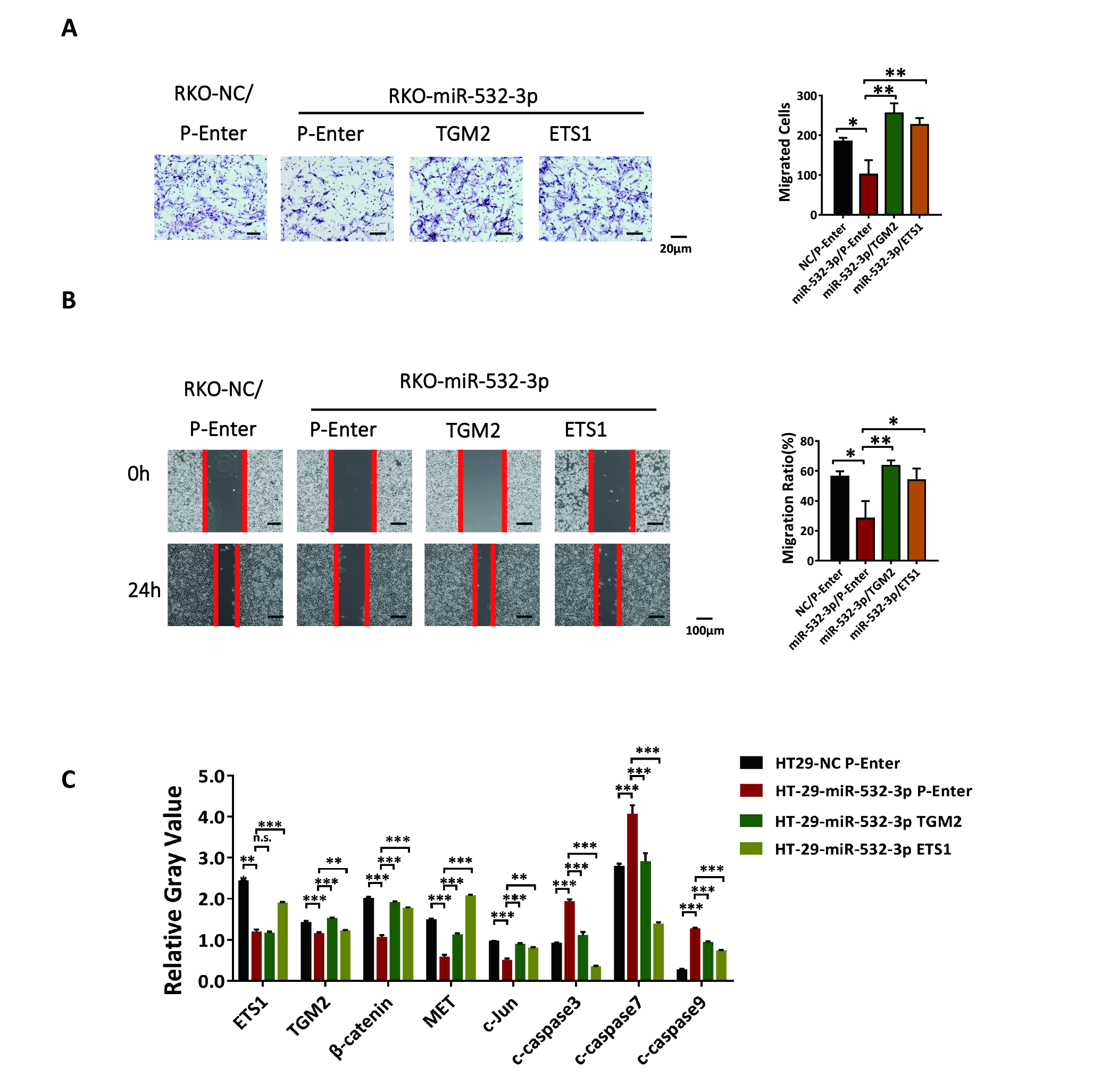

Supplement: Supplementary file 4 — Supplementary figure 3 [file 41419_2019_1962_MOESM4_ESM.tif]
