# Supplementary material for: MiR-532-3p suppresses colorectal cancer progression by disrupting the ETS1/TGM2 axis-mediated Wnt/β-catenin signaling
Source: Cell Death Dis. 2019 Sep 30;10(10):739. doi: 10.1038/s41419-019-1962-x (PMC6768886; doi:10.1038/s41419-019-1962-x)
Supplement: Supplementary file 5 — Supplementary Table 1 [file 41419_2019_1962_MOESM5_ESM.docx]

**Supplementary Table 1.** Sequence information used in this study

| siRNA | Sense | antisense |
| --- | --- | --- |
| miR-532-3p mimics | 5'-CCUCCCACACCCAAGGCUUGCA-3' | 5'-CAAGCCUUGGGUGUGGGAGGUU-3' |
| negative control | 5'-UUCUCCGAACGUGUCACGUTT-3' | 5'-ACGUGACACGUUCGGAGAATT-3' |
| miR-532-3p inhibitors | 5'-UGCAAGCCUUGGGUGUGGGAGG-3' |  |
| inhibitors negative control | 5'-CAGUACUUUUGUGUAGUACAA-3' |  |
| Gene | **Forward** | **Reverse** |
| GAPDH | 5'-GGAGCGAGATCCCTCCAAAAT -3' | 5'-GGCTGTTGTCATACTTCTCATGG -3' |
| TGM2 | 5'-AATCCAGAAATCAAGATCCGGA-3' | 5'-CAGGTCCATTCTCACCTTAACT-3' |
| ETS1 | 5’-TTGAAAGCATAGAGAGCTACGA-3’ | 5’-CTCTGAGTCGAAGCTGTCATAG-3’ |
| GAPDH-ChIP | 5’TACTAGCGGTTTTACGGGCG-3’ | 5’-TCGAACAGGAGGAGCAGAGAGCGA-3’ |
| TGM2-ChIP (-1604/-1462) | 5’-GCCATGCGTCACTCAGGT-3’ | 5’-TGGCTGTGAGAAGAATCCC-3’ |
| TGM2-ChIP (-1264/-1161) | 5’-ACCCAGAGCTGCGTGAC-3’ | 5’- ATCAGCAGCAGCACCAG-3’ |
| TGM2-ChIP (-358/-252) | 5’- CCTCTTGACCGACTTCCC-3’ | 5’- ACCCGTAATTGCCCCAT-3’ |
| TGM2-ChIP-(-186/-13) | 5’- GAGCCCGTTTGACCCAG-3’ | 5’- CCGGAGCAAGCTCTACATT-3’ |
